# Maternal Exposure to Acephate Caused Nephrotoxicity in Adult Offspring Rats Mediated by Excessive Autophagy Activation, Oxidative Stress Induction, and Altered Epithelial Sodium Channel and Na^+^/K^+^-ATPase Gene Expression

**DOI:** 10.3390/biology12020162

**Published:** 2023-01-20

**Authors:** Afoua Mufti, Maroua Jalouli, Saber Nahdi, Nizar Tlili, Wadha Alqahtani, Lamjed Mansour, Saleh Alwasel, Abdel Halim Harrath

**Affiliations:** 1 Laboratory of Biotechnology and Biomonitoring of the Environment and Oasis Ecosystems, Faculty of Sciences of Gafsa, Gafsa 2112, Tunisia; 2 Department of Biology, College of Sciences, Imam Mohammad Ibn Saud Islamic University (IMSIU), Riyadh 11623, Saudi Arabia; 3 Department of Zoology, College of Science, King Saud University, Riyadh 11451, Saudi Arabia; 4 Institut Supérieur des Sciences et Technologies de l’Environnement Borj Cédria, Université de Carthage, Hammam chat 2050, Ben Arous, Tunis 1073, Tunisia

**Keywords:** acephate, nephrotoxicity, autophagy, oxidative stress, immunofluorescence, histopathology

## Abstract

**Simple Summary:**

Although continuous exposure to conventional pesticides has been linked to tissue dysfunction among humans and animals, there is little information regarding the environmental disturbances and their harmful effects on fetuses. In the present study, we investigated the anomalies caused by oral acephate exposure on the kidney function in rat offspring *in utero*. Moreover, this harmful organic pollutant has been reported to promote the development of various cell injuries and tissue functional disorders, such as reproductive toxicity and diabetes. In addition, we found that exposure to acephate during pregnancy can alter renal integrity and induce nephrotoxicity in rat offspring. Furthermore, acephate exposure aggravated kidney injury by enhancing oxidative stress, autophagy, apoptosis, and histopathological alterations. As humans experience increased exposure to agrochemicals worldwide, the obtained data will advance the knowledge of how an unfavorable fetal environment can affect the offspring’s health during adulthood.

**Abstract:**

This study examined how maternal exposure to acephate—an organophosphate-based insecticide—affected the renal development in rat offspring during adulthood. Virgin female Wistar rats were randomly allocated to three groups: group 1 (control) received sterile water; groups 2 and 3 were intragastrically exposed to low (14 mg/kg) and high (28 mg/kg) doses of acephate from day 6 of pregnancy until delivery, respectively. Further, the offspring of the adult female rats were euthanized in postnatal week 8. Compared with the controls, the adult rat offspring with exposure to low and high doses of acephate exhibited elevated plasma creatinine and blood urea nitrogen levels. Additionally, immunofluorescence analysis revealed the upregulation of autophagic marker genes (*Beclin-1* and *LC-3*) in the acephate-treated rat offspring, thereby suggesting the induction of an autophagic mechanism. Notably, the increased malondialdehyde level, decreased glutathione level, and decreased superoxide dismutase and catalase activities confirmed the ability of acephate to induce oxidative stress and apoptosis in the kidneys of the rat offspring. This may explain the renal histopathological injury detected using hematoxylin and eosin staining. Furthermore, a reverse transcription polymerase chain reaction revealed that the mRNA expression levels of the Na^+^/K^+^-ATPase and the epithelial sodium channel (ENaC) genes were significantly higher in the kidney of female offspring than that of controls owing to acephate toxicity. However, there was no significant effect of acephate on the expression of *NHE3* in the treatment group compared with the control group. Overall, the present findings suggest that oxidative stress caused by prenatal exposure to acephate causes nephrotoxicity and histopathological alterations in adult rat offspring, likely by actions on renal ENaC and Na^+^/K^+^-ATPase genes as well as the autophagic markers *Beclin-1* and *LC-3*.

## 1. Introduction

Recent epidemiological studies have reported that a lifetime exposure to environmental contaminants increases the risk of developing chronic diseases, such as diabetes, heart disease [1], obesity, neurological disorders [2], reproductive toxicity [3,4,5], and cancers [6,7]. Globally, chemical pollutant exposure has increased by approximately 35% in the last two decades [8], and unhealthy environments cause an estimated 12.6 million deaths every year (WHO, 2016). Among the contaminants that are currently of major concern, pesticides exhibit major and long-lasting effects on organisms and cause several illnesses [9]. Notably, pesticide exposure occurs primarily through the consumption of food (vegetables, cereals, and fruit) and water contaminated with these agrochemical residues [10].

Globally, organophosphates are the most widely used pesticides, and acephate is among the most dangerous organophosphate that still remains in use [11,12]. It is a harmful organic pollutant that stimulates an excessive synthesis of reactive oxygen species (ROS) and promotes the development of various adverse effect disorders, such as diabetes and reproductive toxicity [1,2,3].

Epidemiological studies have recently suggested that *in utero* exposure to environmental disturbances can affect the developing fetus and lead to permanent diseases in adulthood [4,5]. Moreover, metabolic reprogramming caused by the ingestion of food contaminants, such as pesticides, is reported to cause long-term metabolic dysfunction [6]. Indeed, prenatal insecticide exposure has been reported to cause ovarian toxicity, obesity, and neurobehavioral abnormalities in rats and humans [1,7,8,9].

However, to the best of our knowledge, no systematic study has investigated the effect of prenatal intake of organophosphate-based insecticides on kidney function, and this is the first investigation to show how *in utero* acephate exposure affects kidney function in adult rat offspring. Based on our study results, we hope to raise awareness of how ingestion of this insecticide increases risks to kidney health in future generations and to encourage pregnant mothers to change their consumption behaviors.

## 2. Materials and Methods

### 2.1. Animal Treatment

This study’s experimental protocols were approved by the Ethical Committee for the Care and Use of Laboratory Animals at the University of Gafsa (Reference Number: FSG-AE-20-23). Overall, 24 virgin female Wistar albino rats obtained from SIPHAT (Tunisia) (age, 60 days; weight, 210–240 g) were individually housed in a temperature (21 °C) and humidity-controlled room, with an artificially reversed light–dark cycle and access to water and food. These rats were weighed and randomly divided into three treatment groups. The rats in group 1 (*n* = 8) were considered as controls and those in groups 2 and 3 (*n* = 8 rats per group) were administered acephate (purity 98%; Sigma-Aldrich, St. Louis, MO, USA) by gavage from day 6 of gestation, which corresponded to the date of fetal implantation, until delivery. Furthermore, considering the published lethal oral dosage of 50% (LD50) acephate (866 mg/kg) [10] and previous findings about its toxic effects on different biological systems, groups 2 and 3 were administered 14 or 28 mg/kg body weight acephate based on oral LD50 values of 1/60 and 1/30, respectively.

### 2.2. Biological Sample Collection

At postnatal week 8, the offspring were euthanized, and their blood samples were collected in test tubes treated with ethylenediamine tetraacetic acid (EDTA). The plasma was centrifuged (3500 rpm; 10 min; 4 °C) and used to analyze plasmatic creatinine (Ref 08015) and blood urea nitrogen (BUN) (Ref 08021) using assay kits from Biomaghreb (Tunisia) according to the manufacturer’s instructions. Kidney tissues obtained from each group were collected, weighed, washed with phosphate buffer saline, and fixed in neutral buffered formalin (NBF)-treated paraffin for histopathological and immunofluorescence analyses. Furthermore, the remaining tissues were stored at −80 °C (fixed in RNAlater solution) for RT-PCR studies and oxidative stress analysis.

### 2.3. Histological Preparation

The kidney tissues were then cut into consecutive fragments, placed in a fixative (10% NBF) solution for 48 h at room temperature, embedded in paraffin, cut into 5-µm-thick sections, and stained with hematoxylin and eosin (H&E). Under light microscopy, the samples were analyzed by assessing the morphological changes, and some blocks were cut into 3-µm-thick sections for immunofluorescence analyses.

### 2.4. Immunofluorescence Staining and Confocal Microscopy

The immunofluorescence procedures were performed as described in a previous study [8]. After placing them on hotplates (60 °C), the slides of the kidney samples from all experimental groups were dewaxed twice with xylene for 10 min; they were then subsequently rehydrated with decreasing concentrations of ethanol and rinsed twice with H_2_O_2_ and thrice with phosphate buffered saline.

The tissue sections were then placed in a solution containing sodium citrate (0.1%) and Triton X-100 (0.1%). After blocking in 1% BSA in PBS for 20 min at room temperature (37 °C), the slides were incubated overnight at 4 °C with the primary antibody against *LC-3* (1:100 dilutions, Dg-Peptide Co., Hangzhou, China) and *Beclin-1* (1:100 dilutions, Dg-Peptide Co.), followed by incubation in a dark room with antirabbit FITC-conjugated secondary antibody (1:2000 dilutions, Abcam, Boston, MA, USA) for 45 min at room temperature. Furthermore, the sections were treated with TE buffer and PBS before adding the Hoechst solution (diluted at 1:15,000, Hoechst 33342, Life Technologies, Waltham, MA, USA). Fluorescence images were captured using an LSM 800 Confocal Microscope (Zeiss Confocal LSM 800, Zeiss, Oberkochen, Germany). Subsequently, the mean fluorescence intensity for protein expression of *LC-3* and *Beclin-1* was recorded using Zen 3.1 service (ZEN lite, Jena, Germany) and quantified using the GraphPad Prism 9 program (GraphPad Prism 9.4.1. Software) in five randomly selected microscopic fields per specimen.

### 2.5. Examination of Gene Expression by RT-PCR

The kidney tissues were first stored at 80 °C in an RNA stabilization reagent solution (Qiagen, Westburg, Leusden, The Netherlands). We subsequently performed a reverse transcription polymerase chain reaction (RT-PCR) based on a previously described protocol [8]. Total RNA was extracted using the RNeasy Mini Kit (Qiagen, Westburg), and 2-μg RNA was used for the reverse transcription using an iScript™ cDNA synthesis kit (Applied Biosystem, Carlsbad, CA, USA) as per the manufacturer’s instructions.

SYBR green (Thermo Fisher Scientific, Waltham, MA, USA) was used to perform real-time RT-PCR with gene-specific primers (Table 1), and the Applied Biosystems 7500 Fast RT-PCR system (Carlsbad, CA, USA) was used according to the following protocol: 1 cycle of initial denaturation at 95 °C for 2 min followed by 40 cycles at 94 °C for 20 s, 58 °C for 20 s, and 72 °C for 20 s. Gene expression was quantified using the 2^−ΔΔCT^ method, with glyceraldehyde 3-phosphate dehydrogenase as the standard control.

### 2.6. Oxidative Stress Measurement

#### 2.6.1. Kidney Tissue Extract Preparation

The kidney tissues were homogenized with a glass homogenizer into 2 mL of ice-cold lysis buffer (pH = 7.4). The cells in the prepared homogenized solution were lysed using ultrasonic cell disruption and then centrifuged at 13,000 rpm (4 °C) for 15 min to obtain the tissue supernatant. The sample was then frozen at −20 °C in aliquots until analysis.

#### 2.6.2. Measurement of Lipid Peroxidation in Kidney Tissue

The levels of malondialdehyde (MDA) in the kidney tissues were evaluated according to a previously described method [11]. Notably, 600 μL of thiobarbituric acid (TBA) solution (diluted in 20% TCA) was mixed with 200 μL of serum sample to generate 800 μL of MDA–TBA adduct. Furthermore, the sample was incubated at 95 °C for 1 h and placed in an ice bath for 15 min. The absorbance was measured at 532 nm using a spectrophotometer (Shimadzu, UV-1800; Shimadzu Corporation, Kyoto, Japan). Notably, the MDA value was measured as nanomoles of MDA per gram of protein.

#### 2.6.3. Determination of Kidney Enzymatic and Nonenzymatic Antioxidant Activities

The total cellular glutathione (GSH) was estimated as described in a previous study [12], with some modifications. Briefly, a solution containing 1500 µL of Tris-HCl buffer (200 mM), 500 µL of EDTA at a pH of 7.5 (0.2 mM), 100 µL of DTNB (10 mM), and 790 µL of methanol was added to 1500 µL of supernatant. The obtained solution was then vortexed and incubated for 30 min at 37 °C. The absorbance was measured at 412 nm using a spectrophotometer (Shimadzu, UV-1800), and the results were expressed as μmole per g tissue.

Catalase (CAT) activity was calculated as described in a previous study [12], with some modifications. Briefly, 20 µL of renal homogenate was added to 100 µL of potassium phosphate buffer (50 mM; pH 7.0) containing H_2_O_2_ (100 mM). Notably, the optical density was estimated for 120 s at 240 nm. All data were expressed as CAT activity per gram of protein. Superoxide dismutase (SOD) activity was measured according to the SOD assay procedure reported by Marklund and Marklund [13]. Furthermore, 2.15 mL of a solution containing 0.1 mol/L Tris-HCl buffer solution and 1 mmol/L EDTA was added to 0.15 mL of pyrogallol solution (2 mM in 10 mM HCI solution), and the absorbance was measured at 480 nm and 25 °C. In addition, the autoxidation rate of pyrogallol (control) was determined from the slope of the absorbance curve during the initial 1 min of the reaction.

The protein contents in the renal homogenates were determined via the method reported by Bradford [14] using bovine serum albumin as a standard. The protein concentrations were measured by adding Coomassie dye to the sample under acidic conditions. The results were then analyzed using a spectrophotometer at 595 nm, and values were expressed as nM mg protein. Furthermore, the changes in absorbance per minute in the treatment group were compared with those in the control group, and the results were expressed as nM mg protein.

All chemicals used to analyze oxidative stress were purchased from Sigma Chemical Co. (St. Louis, MO, USA) and were of analytical grade or the highest grade available.

### 2.7. Statistics

The results are expressed as the mean ± standard deviation (SD). The Tukey’s test and one-way analysis of variance were used to determine significant differences between the control and treatment groups using GraphPad Prism version 5. Differences were considered statistically significant at a *p*-value of <0.05.

## 3. Results

### 3.1. Histopathological Effect of Acephate on the Kidney Tissue

**Figure 1:**
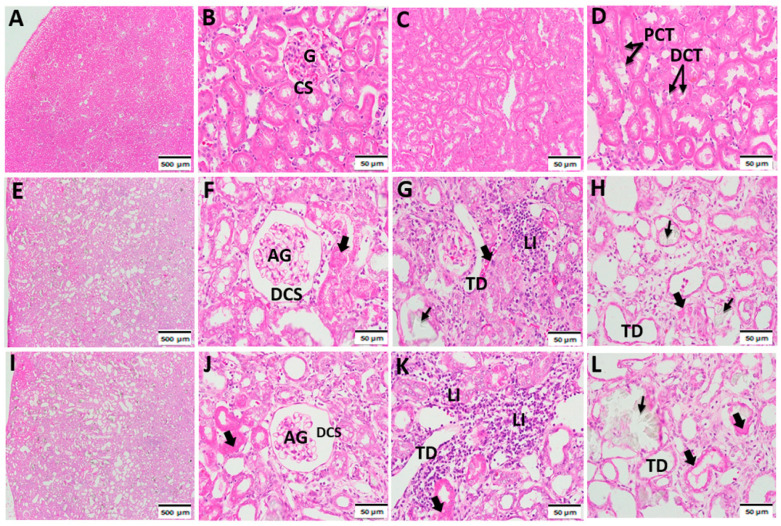
Effects of prenatal acephate exposure on renal histology in adult rat offspring (*n* = 8/group). Kidney sections from the treated groups were stained using hematoxylin and eosin reagents (G × 200). Photomicrographs of the kidneys of the control (**A**–**D**), low-dose acephate (**E**–**H**), and high-dose acephate (**I**–**L**) groups. The control group demonstrated a normal renal cortex structure, with normal glomeruli (G), capsule space (CS), and distal (DCT) and proximal tubules (PCTs). The acephate-treated groups (14 and 28 mg/kg) demonstrated tubular dilatation (TD), calcification (arrow), renal cell fibrosis (large arrow), excessive leukocyte infiltration (LI), and atrophy of glomeruli (AG) with dilatation of CS (DCS).

As previously described, the fixed kidney slices from the control and treatment groups were examined using H&E staining to evaluate histopathological changes (Figure 1).

The kidney tissues from the control groups revealed normal structures. Notably, glomeruli were seen in the kidney cortex sections. Each glomerulus had a bundle of looped capillaries surrounded by narrow Bowman’s spaces, with intact distal and proximal convoluted tubules (Figure 1A–D). Compared with the control group, the kidney sections of the treatment groups revealed noticeable alterations, including a high number of sclerosed glomeruli with periglomerular fibrosis (Figure 1E,F). Additionally, a higher number of foci of calcifications and evidence of inflammation were detected (Figure 1G). Furthermore, a divergence in the simple squamous parietal layers of the Bowman’s capsules was prevalent in the acephate-treated groups in addition to large voids in the capsule space (Figure 1H). The histological analysis of the renal tubules of acephate-treated rat offspring revealed remarkable tubular dilatation and fibrosis as well as a high level of calcification, with inflammatory cell infiltration in the tubular lumen and collecting ducts (Figure 1I–L).

### 3.2. Effect of Acephate Treatment on Plasmatic Creatinine and BUN

**Figure 2:**
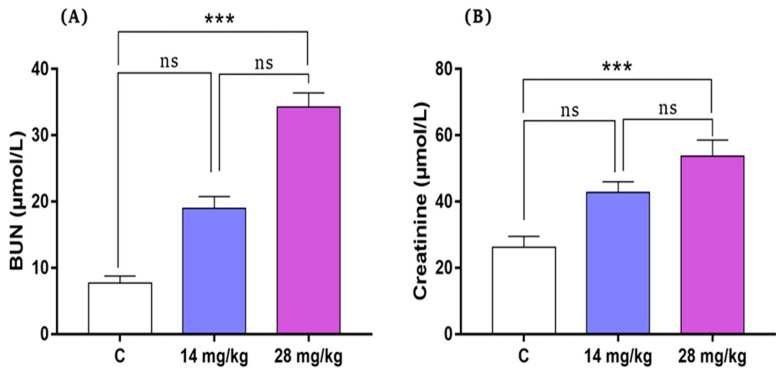
Variations in plasmatic BUN (**A**) and creatinine (**B**) levels in the control and treatment group rats (*n* = 8/group). Values are expressed as mean ± standard deviation (SD). *** *p* < 0.001, significant differences compared with controls. BUN, blood urea nitrogen; ns, non-significant.

Figure 2 shows plasmatic creatinine and BUN levels in all rats from the treatment groups. Notably, the low-dose administration of acephate (14 mg/kg) did not affect the concentration of these two biochemical parameters. However, prenatal exposure to high-dose acephate (28 mg/kg) significantly increased the plasmatic creatinine and BUN levels in the rat offspring compared with the controls (*p* ˂ 0.001).

### 3.3. Analysis of Autophagic Markers in Kidney Tissue

**Figure 3:**
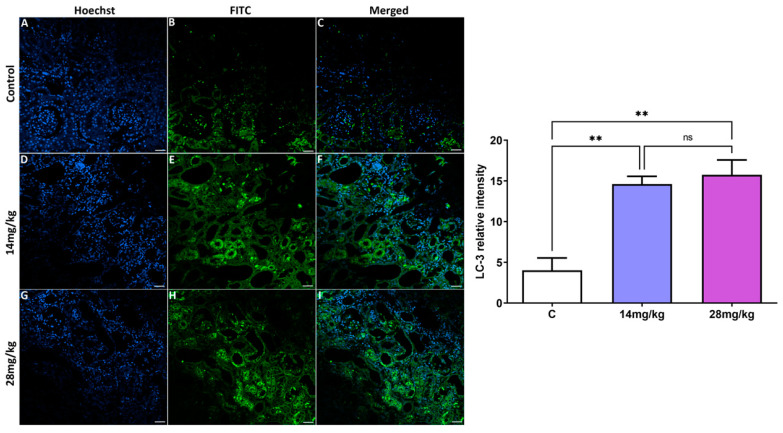
Effects of prenatal acephate exposure on the autophagic marker *LC-3* in adult rat offspring. The autophagic marker LC-3 was evaluated via immunofluorescence staining and measurement of the relative fluorescence intensity (**A**–**I**), which was then quantified using Zen 3.1 service (ZEN lite) and the GraphPad Prism 9 program. An immunofluorescence analysis of *LC-3* revealed the upregulation of this biomarker in both treatment groups (14 and 28 mg/kg) compared with the control group. All data are expressed as mean ± standard deviation (SD). Scale bar = 200 µm. ** *p* < 0.01 (*n* = 8), ns: nonsignificant.

**Figure 4:**
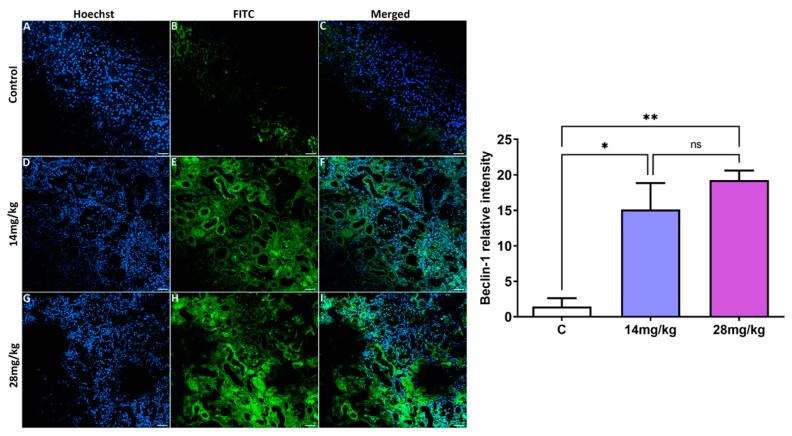
Effects of prenatal acephate exposure on the autophagic marker *Beclin-1* in adult rat offspring. Beclin-1 was evaluated via immunofluorescence staining (**A**–**I**) and measurement of the relative fluorescence intensity, which is then quantified using the Zen 3.1 service (ZEN lite) and quantified using the GraphPad Prism 9 program. The immunofluorescence analysis of Beclin-1 revealed the upregulation of this biomarker in both treatment groups (14 and 28 mg/kg) compared with the control group. All data are expressed as mean ± standard deviation (SD). Scale bar = 200 µm. * *p* < 0.05; ** *p* < 0.01 (*n* = 8), ns: nonsignificant.

Figure 3 and Figure 4 show the expression of LC-3 and Beclin-1 proteins (A–I) from the kidney tissue of the adult offspring, as evaluated by immunofluorescence staining of the three experimental groups. Acephate administration (14 and 28 mg/kg) in the two treatment groups upregulated the expression of LC-3 and Beclin-1 proteins compared with the control group. The effect of acephate administration on the autophagy genes was further confirmed by quantitative analysis (Figure 3 and Figure 4), which indicated that *LC-3*- and *Beclin-1*-related intensities were markedly increased after acephate administration (*p* ˂ 0.01). In addition, the increased *LC-3* and *Beclin-1* levels indicate the occurrence of increased autophagy in the kidneys of both treatment groups. However, there was no significant difference in changes in the expression levels between the groups treated with high (28 mg/kg) and low (14 mg/kg) doses of acephate.

### 3.4. Gene Expression Using RT-PCR

#### 3.4.1. The Epithelial Sodium Channel (ENaC)

**Figure 5:**
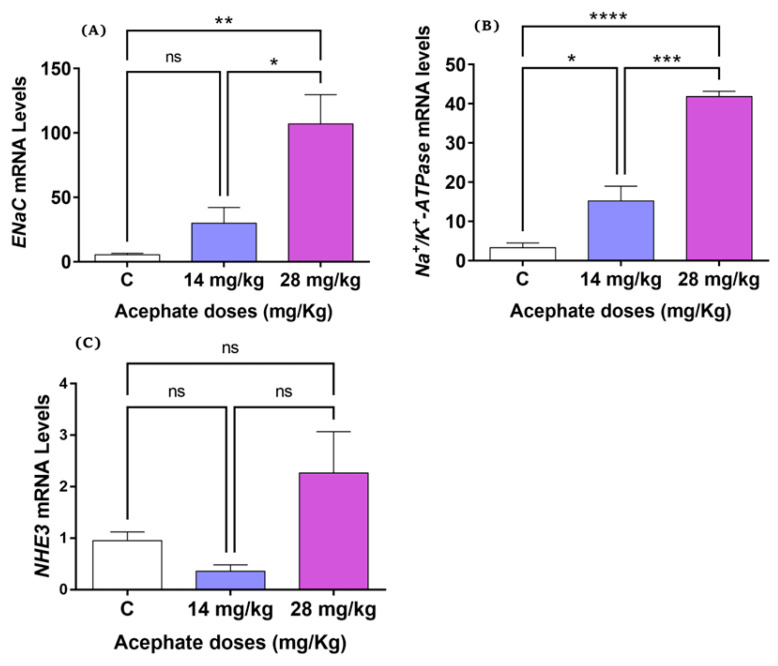
Effect of prenatal acephate exposure on the expression of genes involved in renal toxicity in adult rat offspring. The mRNA expression levels of the genes *ENaC* (**A**), *Na^+^/K^+^-ATPase* (**B**), and *NHE3* (**C**) were analyzed by RT-PCR. All data are expressed as mean ± SD. * *p* < 0.05; ** *p* < 0.01; *** *p* < 0.001; **** *p* < 0.0001, ns: nonsignificant. ENaC, epithelial sodium channel; NHE3, Na^+^/H^+^ Exchanger 3 (*n* = 8).

Figure 5A shows ENaC mRNA levels in the kidney tissue of the study groups. Compared with controls, no significant changes were seen in the ENaC mRNA of the rats treated with low-dose acephate (14 mg/kg); however, the high-dose acephate group (28 mg/kg) demonstrated a significantly higher increase in the levels of ENaC mRNA in female rat offspring (*p* ˂ 0.01) compared with the control group.

#### 3.4.2. The Enzyme Na^+^/K^+^-ATPase

Compared with the control group, the oral administration of acephate at the doses of 14 and 28 mg/kg resulted in the significant upregulation in *Na^+^/K^+^-ATPase* levels in adult offspring, as indicated by the increased mRNA level of this gene in a dose-dependent manner (Figure 5B).

#### 3.4.3. NHE3

Figure 5C shows the levels of *NHE3 mRNA* in all treated animals analyzed by RT-PCR. No significant changes were seen in the expression of this gene within any of the groups.

### 3.5. Effect of Acephate on Renal Oxidative Markers

**Figure 6:**
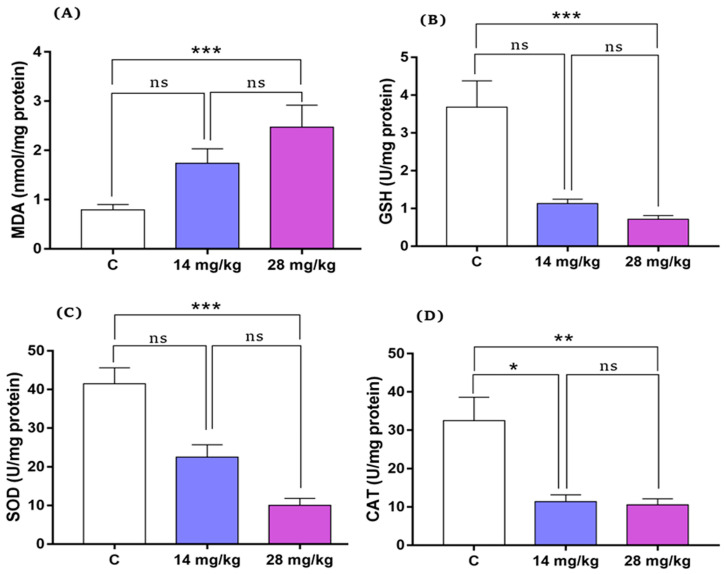
Changes in MDA (**A**) and GSH (**B**) levels and activities of SOD (**C**) and CAT (**D**) in the kidney tissue of adult rat offspring. All data are expressed as mean ± standard deviation (SD). * *p* < 0.05; ** *p* < 0.01, *** *p* < 0.001, ns: nonsignificant. MDA, malondialdehyde; and GSH, glutathione; SOD, superoxide dismutase; CAT, catalase (*n* = 8).

Figure 6 shows levels of antioxidant enzymes (SOD, GSH, and CAT) and MDA in the kidney tissue of the experimental groups. Low-dose acephate (14 mg/kg) treatment showed no significant effect on the levels of MDA, SOD, or GSH in rat offspring; however, CAT activity of the low-dose acephate group was decreased compared with that of the control group (*p* ˂ 0.01).

High-dose acephate (28 mg/kg) treatment caused a significant increase in MDA levels (*p* < 0.001) (Figure 6A) and reduced activity of GSH, CAT, and SOD (*p* < 0.001) compared with the control group (Figure 6B–D).

## 4. Discussion

Although continuous exposure to conventional pesticides has been linked to tissue dysfunction among humans and animals [15,16], there is little information regarding the environmental disturbances and their harmful effects on fetuses. In the present study, we examined abnormalities in the renal function of female rat offspring resulting from the in utero effect of an oral exposure to 14 or 28 mg/kg acephate.

It is well documented that exposure to adverse factors in early life has potential effects on health in adulthood and the incidence of later life adult—onset diseases [17,18]. Previous studies have shown that exposure to acephate decreases the weight of kidney and content of total soluble protein, and induces DNA damage while leading to a dramatic impact of acephate on adult kidney health [19]. However, large gaps remain in revealing the offspring’s developmental toxicity and mechanisms triggered by this organophosphate (OP) insecticide. As expected, our results revealed that in utero exposure to acephate caused histopathological changes revealed by an increase in sclerosed glomeruli, severe tubular fibrosis and dilatation, and leukocyte infiltration. Our data on kidney markers, including creatinine and urea levels, that were affected under acephate confirms the histological data. In fact, it has previously been reported that the release of such specific diagnostic markers is caused by an impaired membrane permeability [20] which is suggested to be caused by an oxygen radical attack of the lipids and proteins of the membrane [21]. Thus, renal dysfunction is mostly associated with oxidative stress, which is most commonly presented as Fanconi’s syndrome, as described in previous studies [22]. Notably, excessive ROS production induces an imbalance between oxidation and antioxidation, leading to oxidative stress, and recent studies have reported the implications of oxidative stress in the development of kidney injury [23,24]. In our study, prenatal acephate exposure caused a remarkable increase in the oxidative stress parameters revealed by an increased MDA level and a reduced activity of GSH, CAT, and SOD thereby promoting renal pathologies [24]. These results are in agreement with previous data which also has been reported in rats prenatally exposed to other pesticides [25,26], suggesting that overexposure to chemicals or pollutants results in the endogenous inhibition of antioxidants and promotes kidney tissue damage and renal pathologies [24]. Thus, the up-regulation of MDA and the down-regulation of GSH, CAT and SOD contents, together with histopathological damage, illustrated that the oxidative stress induced by acephate exposure in early life led to renal structural disorders and dysfunction in female offspring during adulthood.

Similarly, we focused on the expression of autophagy-related proteins in the kidney of offspring female rats. This process is a fundamental cell survival mechanism for eliminating dysfunctional proteins and organelles [27], and it is vital for maintaining cellular homeostasis [28]. Under normal conditions, autophagy leads to a reduced apoptotic process in response to stress [29]. However, abnormal/excessive autophagy can accelerate apoptotic cell death [30,31]. Moreover, environmental pollutants can simultaneously cause autophagic flux and cell apoptosis as a protective mechanism [32,33].

*Beclin-1* promotes the formation of autophagosomes and starts the process of autophagy [34], whereas the mammalian autophagy protein, microtubule-associated protein light chains 3 (LC3), is a marker of autophagosomes that is used to monitor the number of autophagosomes and autophagic activity. According to our results, in utero exposure to excessive acephate increases the levels of *LC-3* and *Beclin-1* genes, leading to the induction of autophagy in the kidneys of rat offspring, similar to the obtained results on the involvement of autophagy in kidney injury through the upregulation of *LC3* and *Beclin* [35,36,37]. The increased autophagy that was accompanied by an increased MDA and decreased GSH levels as well as reduced SOD, GPx, and CAT activities, suggests an oxidative stress-induced autophagy in the acephate-treated kidney. In fact, oxidative stress has a fundamental role in autophagy and apoptosis induction. Similar to our results, Guo et al. [38] have reported that oxidative stress-induced autophagy and apoptosis were involved in NiCl2-induced nephrotoxicity [38]. Furthermore, Caglayan et al. also have demonstrated that cyclophosphamide treatment activated the autophagic pathway by increasing LC3 expression and also the expression of the marker of oxidative DNA damage, 8-hydroxy-2′-deoxyguanosine (8-OHdG), indicating the oxidative stress-induced autophagy in the cyclophosphamide—treated kidney [39].

It has been demonstrated that the control of extracellular fluid volume is associated with the regulation of key sodium transporters—such as ENaC—in the kidney tubules [40,41]. This channel regulates salt reabsorption and plays a key role in osmolarity and blood pressure regulation [42]. Our study reported increased ENaC expression in the kidney tissue of the acephate-treated groups, suggesting that prenatal exposure to acephate alters renal structure or function. However, the molecular mechanism that explains how acephate activates ENaC expression remains unclear. Similar findings have been previously reported [43,44], demonstrating that many chemical structure-treated rats had ENaC activation in the renal tubular cells, which resulted in kidney function impairment. Similarly, we found that the membrane protein Na^+^/K^+^-ATPase is expressed in high levels in the kidneys and plays a vital role in kidney function regulation by extruding intracellular sodium into the interstitial space [45]. Moreover, sodium concentration strongly determines osmotic balance and is useful for filtering wastes in the blood and reabsorbing amino acids. Duchnowicz et al. reported that Na^+^/K^+^-ATPase activity might be affected by endogenous and exogenous factors [46]. In the present study, we found a remarkable elevation in the gene expression of Na^+^/K^+^-ATPase in the kidney tissues in acephate-treated rat offspring. This upregulation might play an essential role in the pathophysiology of renal failure.

The sodium–hydrogen exchanger 3 (NHE3) is another factor that plays a central role in sodium homeostasis; it mediates electroneutral exchange of Na^+^ for H^+^ [47]. In mammals, these transmembrane proteins mediate both HCO^3−^ absorption and H^+^ excretion in the renal tubules and are thereby involved in acid–base homeostasis. Our results showed that prenatal exposure to acephate had no effect on NHE3 gene expression at either low or high concentrations. This phenomenon suggested an increase in the activity or binding affinity of the NHE3 gene, despite the unchanged expression in the kidneys of the offspring. Another possible explanation is that a post-transcriptional event caused post-Na^+^/H^+^ exchange activity and NHE3 protein abundance in renal proximal tubules, although NHE3 mRNA was unaffected [48].

Briefly, our study results reveal that oxidative stress related to *in utero* acephate exposure increases autophagy, apoptosis, and histopathological injury. We found that actions on the *Beclin-1* and *LC-3* genes as well as the disruption of Na^+^/K^+^-ATPase and ENaC enzyme levels cause this injury. For the well-being of future generations, we need to understand the effects of in utero acephate exposure on kidney health and how each of the different processes (oxidative stress, autophagy, and apoptosis) controls the others. Further *in vitro* and *in vivo* studies should be considered to understand how insecticides alter the renal mechanism(s) and the corresponding signaling pathway(s).

## 5. Conclusions

To the best of our knowledge, the study is the first to provide evidence that prenatal exposure to acephate can alter renal integrity and induce nephrotoxicity in rat offspring. Acephate administration significantly altered the mRNA expression levels of the ENaC and Na^+^/K^+^-ATPase genes, promoting kidney function failure and histopathological alterations. Additionally, acephate administration increased the levels of autophagic markers *Beclin-1* and *LC-3*. Moreover, acephate exposure increased MDA and GSH levels and reduced SOD, CAT, and GPx activities. The oxidative stress related to prenatal acephate exposure caused kidney injury, likely through the actions on renal ENaC and Na^+^/K^+^-ATPase genes as well as the autophagic markers. However, further studies are needed to test this hypothesis. Given the high global human exposure to agrochemicals, our data are relevant in understanding how an unfavorable fetal environment can affect the offspring’s health in adulthood.

## Figures and Tables

**Table 1:** Primers for real-time RT-PCR.

Primer	F	R
NHE3	GGAACAGAGGCGGAGGAGCAT	GAAGTTGTGTGCCAGATTCT
ENaC	TACCCTAAGCCCAAGGGAGT	TGTTCTGCAAGGACAGCATC
Na^+^:K^+^ ATPase	TGCCTTCCCCTACTCCCTTCTCATC	CTTCCCCGCTGTCGTCCCCGTCCAC
Glyceraldehyde 3-phosphate dehydrogenase	TCCCTCAAGATTGTCAGCAA	AGATCCACAACGGATACATT

## Data Availability

Not applicable.
